# Pica associated with iron deficiency or depletion: clinical and laboratory correlates in 262 non-pregnant adult outpatients

**DOI:** 10.1186/1471-2326-10-9

**Published:** 2010-12-22

**Authors:** James C Barton, J Clayborn Barton, Luigi F Bertoli

**Affiliations:** 1 Southern Iron Disorders Center, Birmingham, Alabama, USA; 2 Department of Medicine, University of Alabama at Birmingham, Birmingham, Alabama, USA; 3 Department of Medicine, Brookwood Medical Center, Birmingham, Alabama, USA; 4 Brookwood Biomedical, Birmingham, Alabama, USA

## Abstract

**Background:**

There are many descriptions of the association of pica with iron deficiency in adults, but there are few reports in which observations available at diagnosis of iron deficiency were analyzed using multivariable techniques to identify significant predictors of pica. We sought to identify clinical and laboratory correlates of pica in adults with iron deficiency or depletion using univariable and stepwise forward logistic regression analyses.

**Methods:**

We reviewed charts of 262 non-pregnant adult outpatients (ages ≥18 y) who required treatment with intravenous iron dextran. We tabulated their sex, age, race/ethnicity, body mass index, symptoms and causes of iron deficiency or depletion, serum iron and complete blood count measures, and other conditions at diagnosis before intravenous iron dextran was administered. We excluded patients with serum creatinine >133 μmol/L or disorders that could affect erythrocyte or iron measures. Iron deficiency was defined as both SF <45 pmol/L and TS <10%. Iron depletion was defined as serum ferritin (SF) <112 pmol/L. We performed univariable comparisons and stepwise forward logistic regression analyses to identify significant correlates of pica.

**Results:**

There were 230 women (184 white, 46 black; ages 19-91 y) and 32 men (31 white, 1 black; ages 24-81 y). 118 patients (45.0%) reported pica; of these, 87.3% reported ice pica (pagophagia). In univariable analyses, patients with pica had lower mean age, black race/ethnicity, and higher prevalences of cardiopulmonary and epithelial manifestations. The prevalence of iron deficiency, with or without anemia, did not differ significantly between patients with and without pica reports. Mean hemoglobin and mean corpuscular volume (MCV) were lower and mean red blood cell distribution width (RDW) and platelet count were higher in patients with pica. Thrombocytosis occurred only in women and was more prevalent in those with pica (20.4% vs. 8.3%; p = 0.0050). Mean total iron-binding capacity was higher and mean serum ferritin was lower in patients with pica. Nineteen patients developed a second episode of iron deficiency or depletion; concordance of recurrent pica (or absence of pica) was 95%. Predictors of pica in logistic regression analyses were age and MCV (negative associations; p = 0.0250 and 0.0018, respectively) and RDW and platelet count (positive associations; p = 0.0009 and 0.02215, respectively); the odds ratios of these predictors were low.

**Conclusions:**

In non-pregnant adult patients with iron deficiency or depletion, lower age is a significant predictor of pica. Patients with pica have lower MCV, higher RDW, and higher platelet counts than patients without pica.

## Background

Pica is the daily compulsive eating of food or non-food items not part of one's habitual diet or preferences. Pica is a distinctive but poorly understood accompaniment of iron deficiency or depletion in some adults, although most pica items contain little or no iron. Hippocrates wrote that "a craving to eat earth" was associated with "corruption of the blood" [1,2]. In the early 15^th ^C, de Cervantes reported a history in which "women that by caprice eat soil, plaster, coal and other disgusting substances" [3]. Physicians of the 19^th ^C reported that persons with chlorosis (predominantly women) had "various forms of pica or morbid appetite, as for pickles, magnesia, cinders, &c" [4], or "capricious appetite" [5]. Individual adults with pica associated with iron deficiency or depletion typically ingest only one or a few substances in a compulsive manner. Pica items are diverse, and vary according to race/ethnicity, culture, and geographic location [1,6-10].

Many reports describe substances that patients with iron deficiency consumed in a compulsive manner, and effects of treatment of iron deficiency on pica [1,4,6-12]. There are few reports in which observations available at diagnosis of iron deficiency were evaluated using multivariable analyses to identify possible significant differences between persons who did and did not develop pica. In a case-control study from France, a logistic regression model demonstrated that iron deficiency and being non-European were significant independent predictors of pica [9]. Significant race/ethnicity differences in the prevalence of pica have also been reported in other iron deficiency case series [8,11,13-15]. Among iron-deficient adults of the same race/ethnicity, some develop pica and others do not [8,11,13-15]. These observations suggest that heritable traits could contribute to pica susceptibility in adults with iron deficiency. It is generally accepted that some patients who developed pica with an initial episode of iron deficiency will develop pica during subsequent episodes of iron deficiency [13,16,17]. Regardless, we were unable to identify reports in which the concordance of pica or no pica with successive episodes of iron deficiency in the same series of patients was quantified. High concordance also suggests that heritable factors increase the likelihood that pica would accompany iron deficiency.

We sought to identify clinical and laboratory correlates of pica in 262 consecutive non-pregnant adult outpatients at diagnosis of iron deficiency or depletion. These patients were referred because they needed treatment with intravenous iron. In each case, we tabulated sex, age, race/ethnicity, body mass index, symptoms attributed to iron deficiency or depletion, causes of iron deficiency or depletion, serum iron and complete blood count (CBC) measures, and other medical conditions at diagnosis of iron deficiency before intravenous iron was administered. We performed univariable and multivariable analyses to identify significant positive and negative predictors of pica. We compared and contrasted our results with previous reports of pica associated with iron deficiency, and discuss abnormalities that could account for pica in iron deficiency and depletion.

## Methods

### Patient selection

The performance of this study was approved by the Institutional Review Board of Brookwood Medical Center. We performed retrospective reviews of the charts of all adult outpatients (≥18 years of age) who were treated with intravenous iron dextran in a single referral hematology and medical oncology practice during the interval 2002-2009. Cases were identified by computerized and manual searches of practice medical and billing records for International Classification of Diseases (ICD) code 280.0 (iron deficiency), ICD code 307.52 (pica), and procedure codes corresponding to administration of intravenous iron dextran. Each patient was evaluated by either JCB or LFB. Each patient was treated with intravenous iron dextran (Imferon^® ^or INFed^®^; Watson Pharma, Inc., Morristown, NJ) because he/she could not tolerate oral iron supplements; his/her iron deficiency or depletion did not resolve with trials of oral iron supplementation; or he/she had anemia or other manifestation(s) too severe to manage with oral iron supplements [8].

We excluded patients who were pregnant; were hospitalized; had serum creatinine >133 μmol/L; had been treated with erythrocyte transfusion to alleviate anemia; had types of acquired anemia other than that due to iron deficiency or depletion; had erythrocytosis, polycythemia, or other bone marrow disorder not in remission; were receiving anti-cancer chemotherapy or radiation therapy; or had hyperferritinemia due to acute phase reaction, chronic inflammation, liver injury, malignancy, or other cause.

### Laboratory techniques

CBCs were performed using Cell-Dyn^® ^1800 or 1500 automated blood counters (Abbott Laboratories, Chicago, IL). Reference ranges for red blood cells (RBC), mean corpuscular volume (MCV), and red blood cell distribution width (RDW) are 4.20-6.30 × 10^12^/L, 80.0-97.0 fL, and 11.5-14.5%, respectively. Anemia was defined as hemoglobin below these lower reference limits (133 g/L, men; 117 g/L, women) [18]. Thrombocytopenia was defined as platelet count <140 × 10^9^/L; thrombocytosis was defined as platelet count >440 × 10^9^/L. Serum iron measures were determined using automated clinical laboratory methods. Total iron-binding capacity (TIBC) was defined as the sum of serum iron (SI) concentration and unbound iron-binding capacity. Transferrin saturation (TS) was defined as the quotient of serum iron concentration by TIBC. Iron deficiency was defined as both serum ferritin (SF) <45 pmol/L and TS <10%. Iron depletion was defined as SF <112 pmol/L.

### Definition of pica

Pica was defined as the daily compulsive eating of food or non-food items, singly or in combination, not ordinarily part of the patient's habitual diet or preferences, for more than one month, and not reasonably attributable to causes other than iron deficiency by the patient or treating physician. We tabulated pica food and non-food items in each case. Most patients were questioned specifically at the time of their initial evaluation for iron deficiency about whether they had pica. Patients whose charts had no report of pica were classified as not having pica.

### Observations for tabulation

We compiled these observations at diagnosis in all eligible patients: age, sex, self-reported race/ethnicity, body mass index (BMI), and presence (or absence) of pica reports. We also recorded and categorized symptoms attributed to iron deficiency or depletion, defined as fatigue (fatigue, weakness, decreased stamina); cardiopulmonary symptoms (shortness of breath, dyspnea on exertion, palpitations, dizziness, syncope); mental manifestations (forgetfulness, slow mentation; difficulty in performing routine mental tasks); epithelial manifestations (angular, cheilosis, glossitis, stomatitis, dysphagia, esophageal web, hair or nail changes, easy bruising); and neuromuscular symptoms (restless legs syndrome (RLS) [19], involuntary muscle contractions, paresthesias).

We grouped causes of iron deficiency or depletion according to these categories: 1) gastrointestinal blood loss, regardless of lesion(s); 2) reproductive tract loss (menses and previous pregnancy); 3) medical losses (laboratory testing, voluntary blood donation, therapeutic phlebotomy, or surgery); 4) chronic malabsorption (gastric bypass surgery, celiac disease, gastric resection); 5) urinary tract blood loss (hematuria); 6) recurrent epistaxis; and 7) unknown cause. One or two predominant causes were recorded in each case according to interpretation of the treating physician and our chart review.

We recorded the presence or absence of some other conditions in each subject because these conditions are common among patients in our practice: cancer in remission (any malignancy other than non-melanoma skin cancer); common variable immunodeficiency (CVID); diabetes mellitus; and *HFE *hemochromatosis associated with C282Y homozygosity. We compiled values of hemoglobin, RBC, MCV, and RDW; lymphocyte, neutrophil, and platelet counts; and SI, TIBC, TS, and SF levels at diagnosis before intravenous iron dextran therapy was administered.

### Pica and recurrent iron depletion or deficiency

In this substudy, we tabulated the presence or absence of pica detected in patients with recurrent iron deficiency or depletion more than six months after resolution of clinical and laboratory abnormalities associated with their respective initial episodes of iron deficiency or depletion.

### Statistical considerations

We reviewed the charts of 267 patients; five cases were excluded because the chart did not contain all variables specified for the present analyses. Thus, the analytic dataset included observations on 262 patients. Continuous variables included age at diagnosis, BMI, CBC values, and serum iron measures. Dichotomous variables included sex, race/ethnicity (white vs. black/African American), presence or absence of pica and other symptoms or conditions attributed to iron deficiency or depletion, causes of iron deficiency or depletion, and history or no history of cancer in remission, CVID, diabetes mellitus, and hemochromatosis. Altogether, there were 32 variables (pica and 31 others). TIBC values >86.8 μmol/L were imputed as 87.0 μmol/L; SF values reported as <4.5 pmol/L were imputed as 2.2 pmol/L. We analyzed the categories anemia, iron deficiency, iron depletion, thrombocytosis, thrombocytopenia, and anemia and iron deficiency in univariable but not multivariable analyses, because each of these categories is derived from measured values. Observations analyzed on 18 persons who had recurrent iron depletion or deficiency included recurrence of pica, CBC values, and serum iron measures.

Descriptive statistics are displayed as enumerations, percentages, frequency distribution plots, or mean ± 1 standard deviation (SD). Comparisons were made using either Student's two-sided t-test or chi-square or Fisher's exact test, as appropriate. We performed stepwise forward multiple logistic regression analyses to identify independent factors that have a significant positive or negative association with pica (dependent variable). Results of the final model are expressed as beta coefficient, standard error (SE), Wald coefficient/SE, probability, and odds ratios (95% confidence interval (CI)). Data analyses were performed using GB-Stat^® ^v 8.0 (Dynamic Microsystems, Inc., Silver Spring, MD) and Microsoft Excel 2000^® ^(Microsoft Corp., Redmond, WA). Values of p < 0.05 are defined as significant.

## Results

### General characteristics of study subjects

All patients were treated with intravenous iron dextran in the interval November 1992-March 2009. There were 230 women (184 white, 46 black) and 32 men (31 white, 1 black). The age range of women was 19-91 years; the age range of men was 24-81 years (Table 1).

**Table 1:** Characteristics of 262 adults with iron deficiency or depletion^1^

Characteristic	White women (184)	White men (31)	Black women (46)	Black men (1)
Age, y	54 ± 16	62 ± 16	42 ± 15	58

Body mass index, kg/m^2^	28.9 ± 7.6	29.0 ± 6.3	32.0 ± 9.4	29.6

Pica, % (n)	42.9 (79)	32.3 (10)	63.0 (29)	0

Fatigue, % (n)	41.3 (76)	41.9 (13)	54.3 (25)	100.0 (1)

Cardiopulmonary symptoms, % (n)	28.8 (53)	38.7 (12)	60.9 (28)	0

Epithelial manifestations, % (n)	26.1 (48)	16.1 (5)	19.6 (9)	0

Mental manifestations, % (n)	6.0 (11)	6.5 (2)	0	0

Neuromuscular symptoms, % (n)	4.9 (9)	6.5 (2)	6.5 (3)	0

				

Gastrointestinal blood loss, % (n)	42.4 (78)	77.4 (24)	17.4 (8)	100.0 (1)

Reproductive blood loss, % (n)	30.4 (56)	-	60.9 (28)	-

Medical blood loss, % (n)	14.1 (26)	9.7 (3)	4.4 (2)	0

Hematuria, % (n)	1.6 (3)	0	0	0

Epistaxis, % (n)	0	6.5 (2)	0	0

Chronic malabsorption, % (n)	12.0 (22)	3.2 (1)	13.0 (6)	0

Unknown cause of iron deficiency/depletion, % (n)	6.5 (12)	9.7 (3)	8.7 (4)	0

				

Hemoglobin, g/L	106 ± 19	105 ± 25	93 ± 20	13.6

MCV, fL	79.5 ± 9.9	78.6 ± 8.2	72.5 ± 16.2	81.0

RBC × 10^12^/L	4.14 ± 0.63	4.16 ± 0.81	3.93 ± 0.69	5.45

RDW, %	17.3 ± 2.8	18.7 ± 3.5	19.3 ± 4.4	16.8

Platelets × 10^9^/L	327 ± 135	264 ± 84	367 ± 125	199

Lymphocytes × 10^9^/L	2.0 ± 0.9	1.8 ± 0.9	2.0 ± 0.9	3.7

Neutrophils × 10^9^/L	4.5 ± 2.2	4.4 ± 2.0	3.6 ± 1.5	3.4

SI, μmol/L	7 ± 5	7 ± 4	8 ± 6	9

TIBC, μmol/L	74 ± 13	70 ± 14	71 ± 14	60

TS, %	10 ± 7	11 ± 8	10 ± 6	15

SF, pmol/L	29 ± 25	36 ± 25	27 ± 22	58

				

Cancer in remission, % (n)	11.4 (21)	22.6 (7)	13.0 (6)	100.0 (1)

CVID, % (n)	21.2 (39)	0	0	0

Diabetes mellitus, % (n)	15.2 (28)	19.4 (6)	13.0 (6)	0

Hemochromatosis, % (n)	2.7 (5)	0	0	0

				

Anemia, % (n)	46.2 (85)	90.3 (28)	93.5 (43)	0

Thrombocytosis, % (n)	14.1 (26)	0	21.7 (10)	0

Thrombocytopenia, % (n)	3.8 (7)	9.7 (3)	0	0

Iron deficiency, % (n)	63.6 (117)	48.4 (15)	69.6 (32)	0

Iron deficiency and anemia, % (n)	46.7 (86)	35.5 (11)	63.0 (29)	0

^1^Results are expressed as mean ± 1 SD or as percentage (n). Abbreviations: MCV, mean corpuscular volume; RBC, red blood cells; RDW, red blood cell distribution width; SI, serum iron; TIBC, total iron-binding capacity; TS, transferrin saturation; SF, serum ferritin; CVID, common variable immunodeficiency.

There were reports of pica in 118 patients (45.0%) (Table 1), and chart documentation of no pica in 49 other cases. Thus, there were explicit reports of pica or no pica in 63.7% of all 262 cases. Among all 262 subjects, the proportion of women with pica did not differ significantly from the proportion of men with pica (47.0% vs. 31.3%, respectively; p = 0.0943). The proportion of whites with reports of pica was lower than the proportion of blacks with pica (41.1% vs. 61.7%, respectively; p = 0.0113). Among women, the proportion of whites with reports of pica was lower than the proportion of blacks with pica (42.9% vs. 63.0%, respectively; p = 0.0145).

Fatigue, cardiopulmonary symptoms, and epithelial manifestations were common among all 262 subjects (43.9%, 35.5%, and 23.7%, respectively). Gastrointestinal blood loss occurred in 42.4% and medical blood loss in 11.8% of all subjects; 36.5% of women had reproductive tract blood loss. Chronic malabsorption occurred in 29 patients (11.2%), 28 of whom (96.6%) were women. Diagnoses of CVID and hemochromatosis were observed only in white women. Thrombocytosis occurred only in women (n = 36; 15.6%) (Table 1).

### Univariable comparisons of subjects with ice pica (pagophagia) and non-ice pica

Epithelial manifestations and chronic malabsorption were less prevalent in 103 patients with pagophagia than in 15 patients with non-ice pica (Table 2). Mean MCV and the prevalence of thrombocytosis were lower in patients with pagophagia than in patients with non-ice pica. Other characteristics did not differ significantly between the two patient groups (Table 2).

**Table 2:** Comparisons of 103 adults with pagophagia and 15 adults with non-ice pica^1^

Characteristic	**Report of pagophagia (n = 103)**^ 2 ^	Pica without report of ice (n = 15)	**Value of p**^ 3 ^
Age, y	49 ± 15	52 ± 18	0.5056

Women, % (n)	90.3 (93)	100.0 (15)	0.2421

White, % (n)	74.8 (77)	80.0 (12)	0.4693

Body mass index, kg/m^2^	29.7 ± 7.6	30.1 ± 11.2	0.8857

Fatigue, % (n)	45.6 (47)	53.3 (8)	0.5764

Cardiopulmonary symptoms, % (n)	42.7 (44)	46.7 (7)	0.7730

Epithelial manifestations, % (n)	26.2 (27)	53.3 (8)	0.0361

Mental symptoms, % (n)	6.8 (7)	0 (0)	0.3758

Neuromuscular symptoms, % (n)	6.8 (7)	0 (0)	0.3758

			

Gastrointestinal blood loss, % (n)	36.9 (38)	33.3 (5)	0.7890

Reproductive blood loss, % (n)	43.7 (45)	20.0 (3)	0.0684

Epistaxis, % (n)	0 (0)	0 (0)	-

Hematuria, % (n)	1.0 (1)	0 (0)	0.8729

Medical blood loss, % (n)	5.8 (6)	20.0 (3)	0.0877

Chronic malabsorption, % (n)	8.7 (9)	40.0 (6)	0.0007

Unknown cause of iron deficiency/depletion, % (n)	8.7 (9)	6.7 (1)	0.7879

			

Hemoglobin, g/L	99 ± 21	110 ± 26	0.2471

MCV, fL	73.8 ± 13.1	82.7 ± 8.9	0.0026

RBC × 10^12^/L	4.16 ± 0.58	4.11 ± 0.95	0.8764

RDW, %	18.7 ± 3.9	17.7 ± 2.3	0.1637

Platelets × 10^9^/L	336 ± 118	464 ± 247	0.0669

Lymphocytes × 10^9^/L	1.8 ± 0.8	2.2 ± 0.7	0.1012

Neutrophils × 10^9^/L	4.2 ± 2.1	4.8 ± 2.0	0.3587

SI, μmol/L	7 ± 5	7 ± 4	0.8412

TIBC, μmol/L	75 ± 13	77 ± 3	0.5761

TS, %	9 ± 8	9 ± 6	0.8805

SF, pmol/L	27 ± 22	27 ± 5	0.8883

			

Cancer in remission, %	8.7 (9)	0 (0)	0.2807

CVID, %	9.7 (10)	20.0 (3)	0.2154

Diabetes mellitus, %	13.6 (14)	0 (0)	0.1319

Hemochromatosis, %	1.0 (1)	0 (0)	0.8729

			

Anemia, % (n)	81.6 (84)	66.7 (10)	0.1808

Thrombocytosis, % (n)	16.5 (17)	46.7 (7)	0.0067

Thrombocytopenia, % (n)	2.9 (3)	6.7 (1)	0.4432

Iron deficiency, % (n)	68.0 (70)	66.7 (10)	0.9201

Iron deficiency with anemia, % (n)	55.3 (57)	40.0 (6)	0.2658

^1^Results are expressed as mean ± 1 SD or as percentage (n). Abbreviations: MCV, mean corpuscular volume; RBC, red blood cells; RDW, red blood cell distribution width; SI, serum iron; TIBC, total iron-binding capacity; TS, transferrin saturation; SF, serum ferritin; CVID, common variable immunodeficiency.

^2^Each of these patients reported pica for ice (pagophagia); some patients reported two or more pica items.

^3^Comparisons were made using Student's two-tailed t-test, Chi-square test, or Fisher's exact test, as appropriate.

### Univariable comparisons of subjects with and without pica

**Figure 1:**
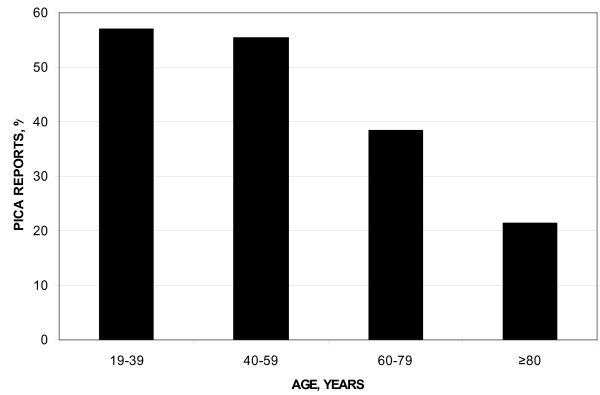
**Frequency distribution plot of percentages of white women with pica and iron deficiency or depletion**. The prevalence of pica reports was greater in women aged 19-59 years than in older women (55.9% vs. 34.8%; p = 0.0061).

Ice was the most common pica item (87.3% of patients with pica reports) (Table 3). Mean age and the prevalence of whites were lower in patients with pica than in patients without pica. The prevalence of pica reports was greater in white women aged 19-59 years than in older women (Figure 1). Cardiopulmonary symptoms and epithelial manifestations were more common in patients who reported pica than in those who did not report pica. Reproductive blood loss was more prevalent in patients with pica reports than in those without pica reports (Table 3). All patients with pica reported that their pica resolved within three weeks (or less) after administration of their first intravenous infusion of iron dextran.

**Table 3:** Comparisons of 118 adults with pica and 144 adults without pica^1^

Characteristic	**Report of pica (n = 118)**^ 2 ^	No report of pica (n = 144)	**Value of p**^ 3 ^
Age, y	50 ± 15	55 ± 17	0.0041

Women, % (n)	91.5 (108)	84.7 (122)	0.0943

White, % (n)	75.4 (89)	87.5 (126)	0.0113

Body mass index, kg/m^2^	29.7 ± 8.1	29.3 ± 7.7	0.6667

Fatigue, % (n)	46.6 (55)	41.7 (60)	0.4224

Cardiopulmonary symptoms, % (n)	43.2 (51)	29.2 (42)	0.0180

Epithelial manifestations, % (n)	29.7 (35)	18.8 (7)	0.0387

Mental symptoms, % (n)	5.9 (7)	4.2 (6)	0.5126

Neuromuscular symptoms, % (n)	5.9 (7)	4.9 (7)	0.7013

			

Gastrointestinal blood loss, % (n)	36.4 (43)	47.2 (68)	0.2411

Reproductive blood loss, % (n)	40.7 (48)	25.0 (36)	0.0068

Epistaxis, % (n)	0	1.4 (2)	0.3011

Hematuria, % (n)	0.8 (1)	1.4 (2)	0.5745

Medical blood loss, % (n)	7.6 (9)	15.3 (22)	0.0564

Chronic malabsorption, % (n)	12.7 (15)	9.7 (14)	0.4429

Unknown cause of iron deficiency/depletion, % (n)	8.5 (10)	6.3 (9)	0.4897

			

Hemoglobin, g/L	100 ± 22	107 ± 22	0.0251

MCV, fL	75.0 ± 13.0	80.8 ± 9.0	0.0001

RBC × 10^12^/L	4.15 ± 0.64	4.08 ± 0.70	0.4232

RDW, %	18.6 ± 3.7	17.2 ± 2.7	0.0006

Platelets × 10^9^/L	352 ± 146	305 ± 113	0.0046

Lymphocytes × 10^9^/L	1.9 ± 0.7	2.0 ± 1.0	0.4120

Neutrophils × 10^9^/L	4.3 ± 2.1	4.3 ± 2.1	0.9327

SI, μmol/L	7 ± 5	8 ± 5	0.1304

TIBC, μmol/L	75 ± 13	72 ± 14	0.0476

TS, %	10 ± 7	11 ± 7	0.1124

SF, pmol/L	27 ± 22	34 ± 25	0.0248

			

Cancer in remission, %	7.6 (9)	18.1 (26)	0.0136

CVID, %	11.0 (13)	18.1 (26)	0.1113

Diabetes mellitus, %	11.9 (14)	18.1 (26)	0.1657

Hemochromatosis, %	0.8 (1)	2.8 (4)	0.2533

			

Anemia, % (n)	79.7 (94)	69.4 (100)	0.0605

Thrombocytosis, % (n)	20.4 (24)	8.3 (12)	0.0050

Thrombocytopenia, % (n)	3.4 (4)	4.2 (6)	0.5031

Iron deficiency, % (n)	55.6 (80)	69.5 (82)	0.0720

Iron deficiency with anemia, % (n)	43.8 (63)	54.2 (62)	0.1495

^1^Results are expressed as mean ± 1 SD or as percentage (n). Abbreviations: MCV, mean corpuscular volume; RBC, red blood cells; RDW, red blood cell distribution width; SI, serum iron; TIBC, total iron-binding capacity; TS, transferrin saturation; SF, serum ferritin; CVID, common variable immunodeficiency.

^2^Pica items included: ice (n = 103); ice water (n = 6); salt (n = 4); sugar (n = 3); dirt, soil, or gravel (n = 3); crackers, laundry starch, pickles, or popsicles (2 each); and candy, cantaloupe, carbohydrates, carbonated beverages, chocolate-covered raisins, coffee beans, dry oatmeal, Gummi^® ^worms, lemons, permanent markers, popcorn, raw cabbage (and broccoli and spinach), and strawberry ice cream (1 each). Some patients reported two or more pica items.

^3^Comparisons were made using Student's two-tailed t-test, Chi-square test, or Fisher's exact test, as appropriate.

Mean hemoglobin and mean MCV values were lower and mean RDW was higher in patients with reports of pica with than in patients without reports of pica (Table 3). Mean platelet counts and prevalence of thrombocytosis were higher in patients with reports of pica. Thrombocytosis occurred only in women and was more prevalent in those with pica (Table 3). Mean TIBC was higher and mean SF was lower in patients with pica reports. The prevalence of iron deficiency, with or without anemia, did not differ significantly between patients with and without pica reports. The prevalence of cancer in remission was lower in patients with pica (Table 3).

Five white women were previously diagnosed to have hemochromatosis, homozygosity for *HFE *C282Y, and iron overload phenotypes. They had been treated with phlebotomy to achieve iron depletion [20]. They subsequently developed iron deficiency and associated symptoms at ages 34-69 years, 2-11 years after achieving iron depletion as part of iron overload management. Their causes of iron deficiency were blood loss at total knee arthroplasty (n = 2); gastrointestinal bleeding from arteriovenous malformations (n = 1); surgery and laboratory blood loss associated with breast cancer treatment (n = 1); and chronic iron malabsorption due to gastric bypass surgery (n = 1). One of these five women reported having pica (Table 3).

### Recurrence of pica

Nineteen patients had recurrent iron deficiency or depletion. Fifteen had pica at first diagnosis of iron deficiency or depletion (13 white women, 1 white man, 1 black woman); 14 of the fifteen (93.3%) had pica with recurrent iron deficiency or depletion. Four other patients (all white women) did not have pica at first diagnosis; none reported pica with recurrent iron deficiency or depletion. Thus, 94.7% of these patients retained the same positive or negative pica phenotype with recurrent iron deficiency or depletion that they had at first diagnosis. The proportions of patients whose pica phenotype was the same at the initial and recurrent episodes of iron deficiency or depletion did not differ significantly (14/15 vs. 4/4, respectively; p = 0.6213, Chi-square test). At recurrence of pica, eight of fifteen subjects (53.3%) had hemoglobin and SF levels that were within the corresponding reference ranges. At recurrence of pica, mean MCV was higher and mean RDW was lower than at first diagnosis of iron deficiency or depletion; other mean CBC values and serum iron measures did not differ significantly (Table 4).

**Table 4:** Characteristics of 14 patients with recurrent pica^1^

Characteristic	First diagnosis	Recurrence	**Value of p**^ 2 ^
Hemoglobin, g/dL	108 ± 16	122 ± 18	0.0500

MCV, fL	75.2 ± 9.9	81.9 ± 7.0	0.0493

RBC × 10^12^/L	4.44 ± 0.48	4.55 ± 0.50	0.5786

RDW, %	18.7 ± 3.2	16.1 ± 2.3	0.0240

Platelets × 10^9^/L	335 ± 87	311 ± 86	0.4901

Lymphocytes × 10^9^/L	1.8 ± 0.7	2.0 ± 0.7	0.3808

Neutrophils × 10^9^/L	4.3 ± 2.0	4.0 ± 1.7	0.6368

SI, μmol/L	7 ± 4	8 ± 4	0.8523

TIBC, μmol/L	75 ± 14	69 ± 8	0.2517

TS, %	8 ± 5	12 ± 6	0.1721

SF, pmol/L	27 ± 22	83 ± 108	0.0824

^1^Each patient had pica at first diagnosis of and at recurrence of iron deficiency or depletion. Data are presented as mean ± 1 SD. Abbreviations: MCV, mean corpuscular volume; RBC, red blood cells; RDW, red blood cell distribution width; SI, serum iron; TIBC, total iron-binding capacity; TS, transferrin saturation; SF, serum ferritin.

^2^Comparisons of mean values at first diagnosis and at recurrence of iron deficiency or depletion were made using Student's two-tailed t-test.

### Multiple logistic regression analyses

We performed stepwise forward multiple logistic regressions using pica as the dependent variable to determine the most economical model that would include only significant explanatory independent variables. We identified four significant predictors of pica among the 31 independent variables: age and MCV (negative associations) and platelet count and RDW (positive associations) (Table 5). No other variable or combination of variables added to this four-variable model achieved statistical significance. The odds ratios and 95% confidence intervals of the four independent variables associated with reports of pica are displayed in Table 5.

**Table 5:** Significant predictors of pica in a logistic regression model^1^

Variable	Beta coefficient	Standard error (SE)	Wald Coefficient/SE	Probability	Odds ratio (exponential beta)	95% Confidence interval of odds ratio
age, y	-0.190	0.009	-2.242	0.025	0.981	(0.965, 0.998)

MCV, fL	-0.024	0.008	-3.119	0.002	0.977	(0.962, 0.991)

RDW, %	0.105	0.032	3.328	0.001	1.111	(1.044, 1.182)

Platelets × 10^9^/L	0.002	0.001	2.288	0.022	1.002	(1.000, 1.004)

^1^These results, obtained in a forward stepwise logistic regression model, include only the significant independent predictors of pica occurrence. Abbreviations: MCV, mean corpuscular volume; RDW, red blood cell distribution width. Significance of this regression: Chi-square (4 degrees of freedom) = 30.42; probability <0.0001.

## Discussion

Pica was the most prevalent symptom we observed (45% of patients). In other studies of adults in the U.S., the prevalence of pica was 36-61% [8,11,13,14]. In contrast, pica was reported in only 0.6% of 353 iron-deficient subjects in Japan [15]. Ice craving (pagophagia) was reported by 87% of the present subjects who reported pica, consistent with the predominance of pagophagia among subjects with pica and iron deficiency in other reports from several countries [1,8,10,11,13,14,16,21,22].

In the present patients with pagophagia, prevalences of epithelial manifestations, chronic malabsorption, thrombocytosis, and mean MCV were lower than in patients with non-ice pica. It is unknown whether differences in choices of pica items are biologically related to these manifestations. The number of patients with non-ice pica was relatively low. Further, studies involving a large number of comparisons have a high likelihood of finding statistically significant associations by chance alone (Type 1 error) [23]. Iron absorption is not significantly affected by pagophagia [24,25], and thus pagophagia is an improbable cause of iron deficiency or depletion.

The proportions of women and men who reported pica did not differ significantly. In two other studies, the prevalence of pica was significantly greater in women than men with iron deficiency [8,14]. In the present women, however, the proportion of whites with pica was significantly lower than that of blacks with pica. In a series of iron deficiency cases from South Africa, there was no significant difference in the prevalence of pica between whites and blacks [10]. We observed that the prevalence of epithelial manifestations was greater in patients with pica reports. In another study, diagnoses of fibromyalgia were significantly more prevalent in iron-deficient subjects with pica than in those without pica [26].

Pica was not significantly associated with the cause(s) of blood loss or iron deficiency or depletion in the present study. This is consistent with previous reports of pica in adults with iron deficiency due to menorrhagia or pregnancy [14]; gastrointestinal blood loss [14]; therapeutic phlebotomy for polycythemia rubra vera [27]; and malabsorption of iron due to gastric bypass [28,29]. Pica is not a predictor of the cause of gastrointestinal blood loss in persons with iron deficiency [14]. Although infection of the stomach with *Helicobacter pylori *was associated with autoimmune gastritis and iron deficiency in men [8,30], there was no significant association of pica with *H. pylori *infection in adults with iron deficiency [25].

Mean hemoglobin levels were significantly lower in the present patients with pica than in those without pica. The prevalence of anemia, iron deficiency, or iron deficiency with anemia did not differ significantly between these two groups. Patients with pica had significantly lower mean values of MCV and significantly higher mean RDW and mean platelet counts than patients without pica. Thrombocytosis was observed only in women with pica, consistent with a previous report of platelet counts in adults with iron deficiency [31,32]. In normal control subjects, mean platelet counts are also significantly higher in women than men [33]. In the present analyses, mean values of TIBC and SF were significantly lower in patients with pica than in patients without pica, but the magnitude of these respective differences was small.

Five white women in the present study were previously diagnosed to have hemochromatosis with homozygosity for *HFE *C282Y and were treated with phlebotomy to achieve iron depletion [20]. Later, they developed iron deficiency due to causes unrelated to therapeutic phlebotomy, like some other patients diagnosed to have *HFE *hemochromatosis in medical care or population screening [34-37]. The proportion of patients with reports of pica who had cancer in remission was lower than the proportion of patients without pica who had cancer in remission. In 55 unselected patients with iron-deficiency anemia due to gastrointestinal blood loss evaluated by a gastroenterology referral service at a city hospital, the proportion of patients with malignancy who had pica was significantly lower than the proportion of patients without malignancy (2/9 vs. 30/46; p = 0.0217; Fisher's exact test) [14].

Using multiple logistic regression analyses, we identified four significant predictors of pica among 31 independent variables: age and MCV (negative associations) and platelet count and RDW (positive associations). Regardless, the odds ratios associated with these variables were relatively low. Only age represents a condition that existed before the development of iron depletion or deficiency in our series of 262 non-pregnant adults. In a French study, age and sex were not significant predictors of pica in a multiple logistic regression model of observations from 79 patients with iron deficiency, although 40% of them were pregnant women [9]. The other "pre-existing" factors that were significant in our univariable comparisons (sex, race/ethnicity, cancer in remission, CVID, diabetes mellitus, and hemochromatosis) were not significant independent predictors of pica in multiple logistic regression analyses. These observations suggest that pica is triggered by factors that precede routine diagnostic indicators of iron depletion or deficiency such as abnormal CBC values or serum iron measures, or structural changes in the epithelial surfaces of the lips, oral cavity, and nasopharynx [38-42].

The present results confirm that pica develops in some adults with iron depletion or iron deficiency and not in others [8,11,13-15], although the reason(s) for this dichotomy is not reported. The concordance of recurrent pica (or absence of pica) in 19 of present patients who developed a second episode of iron deficiency or depletion was high (95%). Further, pica recurred in some of the present patients before they developed anemia or hypoferritinemia. In another study, pica was also a relatively early indicator of recurrent iron deficiency or depletion in patients who had undergone gastric bypass surgery [17]. Other investigators have reported that the occurrence of pica is not necessarily related to the severity of iron deficiency [43]. Thrombopoiesis may be stimulated by early iron depletion [44,45]. Pica in the present patients resolved rapidly after administration of intravenous iron dextran, in agreement with previous reports [4,11,12]. A genetic predisposition to develop pica in the presence of iron deficiency or depletion could explain these observations.

Some reports including the present one suggest that certain manifestations of iron deficiency are affected by heritable traits. *TMPRSS6 *(OMIM *609862; chromosome 22q12-q13) encodes matripase-2 (transmembrane serine protease 6). The *TMPRSS6 *allele A736V (rs855791) was related to significantly lower levels of SI, TS, MCV, and hemoglobin in two genome-wide association studies of twins and general population subjects, respectively [46,47]. *TMPRSS6 *A736V could explain the small but significant differences we observed in mean MCV and hemoglobin levels in patients with and without pica reports, although this is unproven. RLS also occurs as an acquired manifestation of iron deficiency [19,48-50], and is cured by reconstitution of iron stores [51].

Pica ascertainment is an uncertainty in the present study. There was no ascertainment of pica based on chart review in 36.3% of the present cases; patients whose charts had no report of pica were classified as not having pica. Nonetheless, iron depletion or deficiency in all 262 patients in this study was diagnosed and managed by the same two clinicians (JCB and LFB). Accordingly, the likelihood that a distinctive symptom such as pica was not recorded in our medical records, had it occurred, is small. It was beyond the scope of the present study to attempt to contact patients without documented ascertainment of pica for further interview on this point. Uncertainties of our study also include the possibility that some patients did not report pica because they were forgetful or embarrassed [6]. Although the present patients were not treated with oral iron supplements, the proportions of present patients with pica and other manifestations of iron deficiency or depletion are similar to those in reports from North America in which oral iron supplementation was used [8,11,13,14]. Our case series included only one black man, prohibiting us from making conclusions about pica in black men. Medications such as aspirin, non-steroidal anti-inflammatory drugs, and warfarin may increase the loss of blood (and thus iron) from the gastrointestinal tract [52-57]. Other medications, including proton pump inhibitors, histamine receptor-2 antagonists, and preparations that contain calcium, may decrease iron absorption in short-term studies [58-61]. Although we did not tabulate these medications for analysis, other variables such as blood loss from different sites or chronic malabsorption were not significantly associated with increased prevalence of pica.

## Conclusions

In non-pregnant adult patients with iron deficiency or depletion, lower age is a significant predictor of pica. Patients with pica have lower MCV, higher RDW, and higher platelet counts than patients without pica.

## Competing interests

The authors declare that they have no competing interests.

## Authors' contributions

All authors reviewed and edited the manuscript, and all agreed with the form and content of the final manuscript. JCB (first author) designed the study, reviewed patient charts, performed statistical analyses, and drafted the manuscript. JCB (second author) reviewed patient charts, tabulated patient data, and performed statistical analyses. LFB contributed to the study design and reviewed patient charts.

## Pre-publication history

The pre-publication history for this paper can be accessed here:

http://www.biomedcentral.com/1471-2326/10/9/prepub
